# Early-Onset Diabetes Mellitus in Chromosome 8p11.2 Deletion Syndrome Combined With Becker Muscular Dystrophy - A Case Report

**DOI:** 10.3389/fendo.2022.914863

**Published:** 2022-07-25

**Authors:** Conghui Cao, Xiaoli Wang, Xiaojuan Zhao

**Affiliations:** Department of Endocrinology and Metabolism, Institute of Endocrinology, National Health Commission (NHC) Key Laboratory of Diagnosis and Treatment of Thyroid Diseases, The First Affiliated Hospital of China Medical University, Shenyang, China

**Keywords:** chromosome 8p11.2 deletion syndrome, Becker muscular dystrophy, case report, early onset diabetes mellitus, contiguous gene syndrome

## Abstract

**Background:**

Chromosome 8p11.2 includes several key genes in development such as the *FGFR1*, *ANK1*, *KAT6A*, and *SLC20A2* genes. Deletion of this fragment causes a contiguous gene syndrome. Currently, few cases of interstitial deletion of whole 8p11.2 have been reported. We report a rare case of 8p11.2 deletion syndrome with the unique phenotypes, presenting with early-onset diabetes.

**Case Description:**

A 20-year-old man with a 1-year history of diabetes mellitus was admitted to the Endocrinology Clinic. Physical examination revealed the dysmorphic facial features, and broad and foreshortened halluces. Laboratory examination indicated spherocytosis anemia, and hypogonadotropic hypogonadism. Bone mineral density analysis showed decreased bone density in the lumbar vertebrae. Brain CT showed calcification. Whole-exome sequencing revealed a 7.05-Mb deletion in 8p11 containing 43 OMIM genes, and a large in-frame deletion of exons 48–55 in the DMD gene. Metformin was given to the patient after which his blood glucose was well controlled. HCG was injected subcutaneously and was supplemented with calcium and vitamin D, which led to an improvement in the patient’s quality of life.

**Conclusion:**

We report a rare case of 8p11.2 deletion syndrome with unique phenotypes, and early-onset diabetes. It is challenging for endocrinologists to simultaneously reconcile a combination of these diseases across multiple disciplines. We discussed the influencing factors of early-onset diabetes in this patient and speculated that it was caused by complex interactions of known and unknown genetic backgrounds and environmental factors.

## Introduction

Chromosome 8p11.2 includes several key genes in development such as the *FGFR1*, *ANK1*, *KAT6A*, and *SLC20A2* genes. Deletion of this fragment causes a contiguous gene syndrome, including congenital hypogonadotropic hypogonadism (CHH) (1), dysmorphic facial features, hands, and feet (2, 3), spherocytosis (4), Arboleda-Tham syndrome (5), and brain calcification (6). Currently, few cases of interstitial deletion of whole 8p11.2 have been reported. Herein, we report a rare case of 8p11.2 deletion syndrome with the above unique phenotypes, presenting with early-onset diabetes.

## Case Description

**Figure 1:**
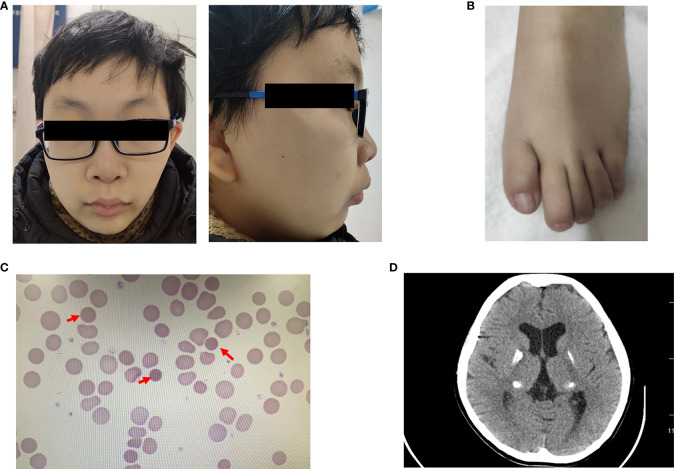
Clinical characters of the patient **(A)** Facial anomalies in the patient, including facial asymmetry, micrognathia, malformed ear, dental dysplasia, and a high-arched palate. **(B)** Feet anomalies showed broad and foreshortened halluces. **(C)** Peripheral blood smear revealed small spherocytes (red arrows). **(D)** Brain CT showed calcification in the bilateral lenticular nucleus, dorsal thalamus, and posterior horn of lateral ventricle.

A 20-year-old man with a 1-year history of diabetes mellitus was admitted to the Endocrinology Clinic of the First Affiliated Hospital of China Medical University. He was born after an uneventful 39-week gestation with a birth weight of 3.0 kg. His parents are non-consanguineous and healthy. His motor skills and language development were delayed. Physical examination revealed the following characteristics: height, 165.0 cm; weight, 50.0 kg; body mass index, 18.36 kg/m2; facial asymmetry; micrognathia; up-slanting palpebral fissure; malformed ear; dental dysplasia; high-arched palate (**Figure 1A**); and broad and foreshortened halluces (**Figure 1B**). Tanner’s staging showed prepubertal signs. A smell test excluded anosmia.

Laboratory examination indicated anemia with a hemoglobin level of 101 g/L. Morphological examination of a peripheral blood smear revealed small spherocytes (**Figure 1C**). Total bilirubin was 71.2 μmol/L. The concentrations of luteinizing hormone and follicle-stimulating hormone were low at baseline and after gonadorelin stimulation, while the testosterone concentration was undetectable. His fasting glucose and insulin levels were 12.12 mmol/L and 9.93 mIU/L, respectively. T1D autoantibodies (GAD, ZnT8, IA-2, ICA, and IAA antibodies) were negative. His HbA1c was 5.8%. Detailed test results are listed in **Tables 1**, **2**.

**Table 1:** Laboratory investigations.

Test	At diagnosis	Follow up (14 months)	Follow up (16 months)	Follow up (20 months)	Normal values
Metabolism					
Body wight (kg)	57	50	51	53	–
PG (mmol/L)	12.12	7.04	5.83	5.81	3.9-6.1
INS (mIU/L)	9.93	8.95	–	5.15	4.03-23.46
CP (pmol/L)	973.3	800.80	–	627.40	99.9-1242.09
HbA1c (%)	5.8	6.4	3.0	3.2	4.4-6
GAD (IU/mL)	6.44	7.20	–	–	0-17
IAA (IU/mL)	27.7	23.2	–	–	0.41-20
GAD	–	–	–	Negative	Negative
IAA	–	–	–	Negative	Negative
ZnT8	–	–	–	Negative	Negative
ICA	–	–	–	Negative	Negative
IA-2	–	–	–	Negative	Negative
LDL-c (mmol/L)	1.47	1.33	–	–	0.00-3.64
TC (mmol/L)	2.67	2.36	–	–	0.00-5.72
TG (mmol/L)	2.06	0.84	–	–	0.00-1.7
HDL-c (mmol/L)	0.54	0.58	–	–	0.91-1.92
UA (umol/L)	291	421	–	301	208-428
LAC (mg/dl)	9.6	–	–	–	4.5-19.8
CK (U/L)	111	–	–	121	50-310
ALT (U/L)	23	29	–	28	9-50
AST (U/L)	17	29	–	26	15-40
LDH (U/L)	168	–	–	–	120-250
**Hematology**					
Hb (g/L)	101	82	89	95	130-175
MCV (fL)	82.5	91.2	89.8	88.5	82.0-100.0
MCC (g/L)	388	346	337	363	316.0-354.0
RET (%)	6.48	9.39	–	–	0.25-1.55
TBIL (μmol/L)	71.2	67.3	–	84.9	0.0-26.0
PLT (109/L)	429	347	376	475	125-350
**Endocrine**					
LH (mIU/mL)	<0.10	<0.10	–	<0.10	0.8-7.6
FSH (mIU/mL)	0.13	0.11	–	0.11	0.7-11.1
E (pmol/L)	36.25	–	–	–	–
T (nmol/L)	<0.69	<0.69	0.70	<0.69	5.54-25.17
FT (pmol/L)	6.56	5.32	7.09	6.32	55.05-183.5
AND (nmol/L)	4.69	3.02	–	–	2.1-10.8
DHEA (umol/L)	3.58	3.61	–	–	2.17-15.2
SHBG (nmol/L)	12.10	11.80	–	–	10-57
PRL (mIU/L)	950.00	1011	–	746	53-360
IGF-1 (ng/ml)	299	294		–	115-358
TSH (mIU/L)	1.78	–	–	–	0.35-4.94
fT4 (pmol/L)	12.60	–	–	–	9.01-19.05
fT3 (pmol/L)	4.66	–	–	–	2.43-6.01
TPOAb (IU/mL)	0.59	–	–	–	0.00-5.61
TGAb (IU/mL)	2.16	–	–	–	0.00-4.11

PG, plasma glucose; INS, serum insulin; CP, serum C peptide; HbA1c, hemoglobin A1c; GAD, glutamic acid decarboxylase; IAA, insulin autoantibody; LDL-c, low density lipoprotein cholesterol; TC, total cholesterol; TG, triglyceride; HDL-c, high density lipoprotein cholesterol; UA, uric acid; LAC, lactic acid; CK, creatine kinase; ALT, alanine transaminase; AST, aspartate transaminase; LDH, lactic dehydrogenase; Hb, hemoglobin; MCV, mean corpuscular volume; MCC, mean corpuscular hemoglobin concentration; RET, reticulocytes; TBIL, total bilirubin; PLT, platelet; LH, luteinizing hormone; FSH, follicle-stimulating hormone; E, Estradiol; T, testosterone; FT, free testosterone; AND, androstendione; DHEA, dehydroepiandrosterone; SHBG, sex-hormone binding globulin; PRL, prolactin; IGF-1, Insulin like growth factor 1; TSH, thyrotropin-releasing hormone; fT4, free thyroxine; fT3, free triiodothyronine; TPOAb, thyroid peroxidase antibody; TGAb, thyroglobulin antibody.

**Table 2:** Results of OGTT and gonadorelin stimulation test.

Oral glucose tolerance test					Normal values
	Fasting	30 min	60 min	120 min	
PG (mmol/L)	12.12	15.71	20.65	25.44	Fasting, 3.9-6.1
INS (mIU/L)	9.93	15.82	18.13	24.52	Fasting, 4.03-23.46
CP (pmol/L)	973.3	1201.70	1393.90	1975.90	Fasting, 99.9-1242.09
**Gonadorelin stimulation test**					
	**Basal**	**30 min**	**60 min**	**120 min**	
LH (mIU/mL)	<0.10	0.49	0.65	0.76	0.8-7.6
FSH (mIU/mL)	0.13	0.64	0.62	0.53	0.7-11.1

PG, plasma glucose; INS, serum insulin; CP, serum C peptide; LH, luteinizing hormone; FSH, follicle-stimulating hormone.

Bioelectrical impedance analysis showed visceral fat of 81 cm^2^, subcutaneous fat of 136.8 cm^2^, and a visceral-to-subcutaneous fat ratio of 59.2%. Testicular ultrasound revealed that the right testis was approximately 1.37 cm × 0.54 cm × 0.93 cm (0.36 ml) in size and that the left testis was approximately 1.29 cm × 0.52 cm × 0.93 cm (0.33 ml) in size. Bone mineral density analysis showed decreased bone density in the lumbar vertebrae (Z = -3.8). Cardiac color Doppler ultrasound indicated that cardiac function and structure were normal, whereas abdominal color Doppler ultrasound showed that the shape and size of the liver were normal, the liver surface was smooth, the edge of the liver was sharp, the echo of liver parenchyma was enhanced, and the contrast of the liver and kidney was increased. The length and diameter of spleen was approximately 10.61 cm, and the thickness of spleen was approximately 5.38 cm, indicating the presence of fatty liver and splenomegaly. Brain CT showed calcification in the bilateral lenticular nucleus, dorsal thalamus, and posterior horn of the lateral ventricle (**Figure 1D**). Ophthalmic examination revealed a cotton wool spot in the right eye retina, which was indicative of stage III diabetic retinopathy. Electromyography was normal, and electroaudiometry indicated normal hearing.

**Figure 2:**
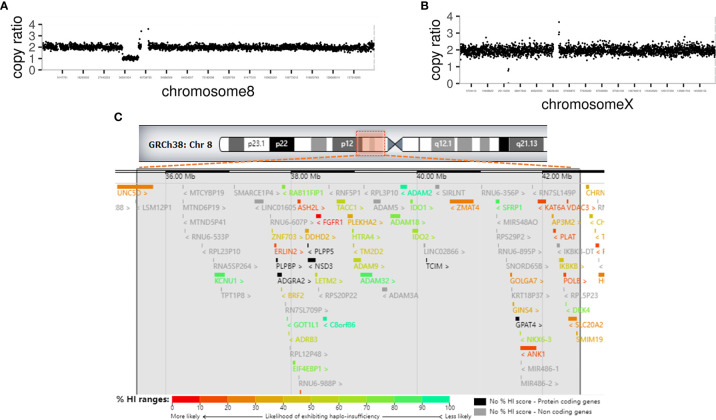
Genetic analysis of the patient **(A)** CNV-seq analysis revealed a large deletion of 7.05 Mb of 8p11 in chromosome 8. **(B)** CNV-seq analysis revealed an in-frame deletion of exons 48-55 in the DMD gene in chromosome X. **(C)** Chromosome ideogram. The impaired chromosomal region is highlighted, a 7.05-Mb region of hemizygous loss in Chr8p11.2 (Chr8: 35541034-42587731). Ninety-four genes are located in the hemizygous loss region of Chr8p11.2, including 43 OMIM genes. Genes are labeled by different colors from unlikely haploinsufficient to likely haploinsufficient, according to DECIPHER (http://decipher.sanger.ac.uk/). High ranks indicate that a gene is more likely to exhibit haploinsufficiency; low ranks indicate a gene is more likely not to exhibit haploinsufficiency.

Whole-exome sequencing revealed a 7.05-Mb deletion in 8p11 (chr8: g.35541034–42587731) containing 43 Online Mendelian Inheritance in Man (OMIM) genes (**Figures 2A, C** and **Table 3**), and a large in-frame deletion of exons 48–55 in the *DMD* gene (chrX: g.31639827–31904558). These abnormalities were confirmed by copy number variation (CNV)-seq analysis (**Figures 2A, B**). Multiplex ligation probe amplification (MLPA)-DMD revealed that the proband’s mother is a heterozygous carrier of the mutant *DMD* gene.

**Table 3:** List of genes in the deletion region.

Gene Symbol	MIM Number for Genes	Phenotype	Inheritance	Phenotypes observed in this patient
UNC5D	616466	–	–	–
KCNU1	615215	–	–	–
ZNF703	617045	–	–	–
ERLIN2	611605	Spastic paraplegia 18, autosomal recessive	AR	–
PLPBP	604436	Epilepsy, early-onset, vitamin B6-dependent	AR	–
ADGRA2	606823	–	–	–
BRF2	607013	–	–	–
RAB11FIP1	608737	–	–	–
GOT1L1	–	–	–	–
ENSG00000285880	–	–	–	–
ADRB3	109691	Obesity, susceptibility to	AD, AR, Mu	Diabetes?
EIF4EBP1	602223	–	–	–
ASH2L	604782	–	–	–
STAR	600617	Lipoid adrenal hyperplasia	AR	–
LSM1	607281	–	–	–
BAG4	603884	–	–	–
DDHD2	615033	Spastic paraplegia 54, autosomal recessive	AR	–
PLPP5	610626	–	–	–
NSD3	607083	–	–	–
LETM2	–	–	–	–
FGFR1	136350	Encephalocraniocutaneous lipomatosis, somatic mosaic	–	–
		Hartsfield syndrome	AD	Malformation of the feet
		Hypogonadotropic hypogonadism 2 with or without anosmia	AD	Hypogonadotropic hypogonadism
		Jackson-Weiss syndrome	AD	Broad and foreshortened halluces
		Osteoglophonic dysplasia	AD	–
		Pfeiffer syndrome	AD	Facial asymmetry, micrognathia, malformed ear, dental dysplasia, and a high-arched palate
		Trigonocephaly 1	AD	–
C8orf86	–	–	–	–
TACC1	605301	–	–	–
PLEKHA2	607773	–	–	–
HTRA4	610700	–	–	–
TM2D2	610081	–	–	–
ADAM9	602713	Cone-rod dystrophy 9	AR	–
ADAM32	618602	–	–	–
ADAM18	619495	–	–	–
ADAM2	601533	–	–	–
IDO1	147435	–	–	–
IDO2	612129	–	–	–
TCIM	607702	–	–	–
ZMAT4	–	–	–	–
SFRP1	604156	–	–	–
GOLGA7	609453	–	–	–
GINS4	610611	–	–	–
GPAT4	608143	–	–	–
NKX6-3	610772	–	–	–
ANK1	612641	Spherocytosis, type 1	AD, AR	Spherocytosis anemia, and splenomegaly
KAT6A	601408	Arboleda-Tham syndrome	AD	Speech delay
AP3M2	610469	–	–	–
PLAT	173370	Hyperfibrinolysis, familial, due to increased release of PLAT	–	–
		Thrombophilia, familial, due to decreased release of PLAT	–	–
IKBKB	603258	Immunodeficiency 15A	AD	–
		Immunodeficiency 15B	AR	–
POLB	174760	–	–	–
DKK4	605417	–	–	–
VDAC3	610029	–	–	–
SLC20A2	158378	Basal ganglia calcification, idiopathic, 1	AD	Brain calcification
SMIM19	–	–	–	–

AD, autosomal dominant; AR, autosomal recessive; MIM, Mendelian Inheritance in Man.

Metformin at 1500 mg/d was given to the patient after which his blood glucose was well controlled. HCG 2000 IU was injected subcutaneously twice a week and was supplemented with calcium and vitamin D, which led to an improvement in the patient’s quality of life.

## Discussion

The deletion range of this patient covers almost the whole 8p11.2 area (chr8: 36,700,001– 43,200,000, 6.5 Mb), which resulted in a haploid deficiency of 94 genes, of which 49 were coding genes (43 OMIM genes); moreover, several genes had high ranks of haploinsufficiency (HI) (**Figure 2C**). Haploinsufficiency of the *FGFR1*, *ANK1*, *KATA6*, and the *SLC20A2* genes causes a rare contiguous gene syndrome that includes CHH with or without anosmia (1), dysmorphic facial features, hands, and feet (Jackson-Weiss syndrome and Pfeiffer syndrome) (2, 3), spherocytosis (4), Arboleda-Tham syndrome (5), and brain calcification (6). These five groups of cardinal signs and symptoms could be named 8p11.2 deletion syndrome. Unlike previous cases reports, in which each patient had 1–3 cardinal symptoms depending on the interstitial deletion length (7–11), the present case developed early-onset diabetes. Interestingly, case no.421734 in decipher database also has diabetes mellitus, whose deletion region is very similar to our case (chr8:36909315-42578314) (12).

Other genes with high ranks of haploinsufficiency (0%–20%) were identified in the deleted region as follows: *UNC5D* (3.53%), *STAR* (11.9%), *POLB* (15.57%), *PLAT* (15.69%), *ERLIN2* (17.19%), *VDAC3* (19.05%), and *ASH2L* (19.21%). Among them, the *UNC5D*, *POLB*, *VDAC3*, and *ASH2L* genes have unknown functions. Biallelic pathogenic variants in the *STAR* gene is associated with lipoid congenital adrenal hyperplasia, which presented with symptoms such as salt wasting, hyponatremia, hypovolemia, hyperkalemia, acidosis, and death in infancy (13). *PLAT* is related to hyperfibrinolysis or thrombophilia with pending confirmation (14). *ERLIN2* is related to autosomal recessive spastic paraplegia-18 (15). None of these genes related to the phenotypes were identified in this patient, which could be attributable to the fact that the haploinsufficiency of these genes is not enough to cause diseases.

In male individuals with CHH, low testosterone levels are strongly associated with an increased risk of developing type 2 diabetes mellitus and osteoporosis (16, 17). Specifically, FGF21/KLB/FGFR1 signaling related CHH is prone to metabolic defects (17). Furthermore, we cannot exclude other genes in this range that might contribute to the development of diabetes; for example, SNPs in the *ADRB3* and *ANK1* genes may be associated with obesity and T2DM (18, 19). However, at present, there is no direct evidence to prove that the deficiency of the *ADRB3* and *ANK1* genes is associated with human diabetes. Only indirect evidence has shown that SNP-induced *ADRB3*-mediated impairment in cAMP production was associated with T2DM susceptibility and development (20). In contrary, *ANK1* gene SNPs causing increased promoter activity and sAnk1 expression in skeletal muscle might contribute to T2DM susceptibility (21). Some evidence also suggests that the *EIF4EBP1* and *SFRP1* genes are involved in mTORC1 signaling in cellular energy metabolism and Wnt signaling in adipogenesis, respectively (22, 23).

Becker muscular dystrophy (BMD, OMIM 300376) is a clinically heterogenous disorder caused by pathogenetic variants in the *DMD* gene. *DMD* is the largest known human gene comprising 79 exons and is inherited in an X-linked recessive manner (24). Patients with distal in-frame deletions showed a milder phenotype than those with deletions proximal to exon 45 (24–26). In particular, severe muscular involvement is reduced in patients sharing in-frame deletion of exons 45–55, and exon-skipping therapy has been developed to convert the severe phenotype to a more benign form (27, 28). The mild BMD phenotype of this patient could also be explained by the frame-shift theory (28). Usually, patients with DMD/BMD have fewer physiological activities, which is also a risk factor for obesity and diabetes (29). Due to the mildness of symptoms, this patient did not require glucocorticoid treatment, which was very fortunate for this patient because he avoided the endocrine problems caused by long-term use of glucocorticoids, such as aggravation of diabetes, osteoporosis, and decreased testosterone levels (30).

All of the above-involved genes are related to type 2 diabetes development but do not cause any known monogenic defects in diabetes. Thus, metformin will be the first choice for patients with 8p11.2 deletion syndrome with diabetes.

In addition, HbA1c levels in patients with hereditary spherocytosis are lower (31). When the patient’s blood sugar was high, the HbA1c was 5.8%, and after reasonable blood sugar control, the HbA1c was 3.0-3.2%. Glycosylated albumin and peripheral glycemic measurements are required to monitor this patient’s glycemic control.

Here, we report a rare case of 8p11.2 deletion syndrome with unique phenotypes involving developmental, hematological, endocrine, metabolic changes, and early-onset diabetes. It is challenging for endocrinologists to simultaneously reconcile a combination of these diseases across multiple disciplines. We discussed the influencing factors of early-onset diabetes in this patient and speculated that it was caused by complex interactions of known and unknown genetic and environmental factors.

## Data Availability Statement

The datasets for this article are not publicly available due to concerns regarding participant/patient anonymity. Requests to access the datasets should be directed to the corresponding author.

## Ethics Statement

The studies involving human participants were reviewed and approved by First Affiliated Hospital of China Medical University. The patients/participants provided their written informed consent to participate in this study. Written informed consent was obtained from the individual(s) for the publication of any potentially identifiable images or data included in this article.

## Author Contributions

Study design, XW. Data collection, CC and XZ. Manuscript drafting, CC and XW. Data interpreting, XW. Revision of the manuscript, XZ and XW. Approval of final version of the manuscript, XW, CC, and XZ. All authors contributed to the article and approved the submitted version.

## Funding

Sinocare Diabetes Foundation (2020SD05).

## Conflict of Interest

The authors declare that the research was conducted in the absence of any commercial or financial relationships that could be construed as a potential conflict of interest.

## Publisher’s Note

All claims expressed in this article are solely those of the authors and do not necessarily represent those of their affiliated organizations, or those of the publisher, the editors and the reviewers. Any product that may be evaluated in this article, or claim that may be made by its manufacturer, is not guaranteed or endorsed by the publisher.
